# Cerebral Venous Sinus Thromboses in Young Stroke Patients: A Case Study to Highlight the Importance of Venous Imaging

**DOI:** 10.7759/cureus.91762

**Published:** 2025-09-07

**Authors:** Megan R Sambrook Smith, Hla Hla Aye, Eluzai Hakim

**Affiliations:** 1 Department of Stroke Medicine, University Hospitals Dorset NHS Foundation Trust, Bournemouth, GBR; 2 Department of Acute Medicine, Royal Bournemouth Hospital, Bournemouth, GBR

**Keywords:** atypical stroke, case report, cerebral venous sinus thrombosis (cvst), ct and mr venography, delayed diagnosis, stroke in young patients, therapeutic anticoagulation, venous stroke

## Abstract

Cerebral venous sinus thrombosis (CVST) is a rare but potentially treatable cause of stroke, often affecting younger individuals and presenting with non-specific symptoms such as headache, seizures, or altered consciousness. Timely diagnosis is essential but is frequently delayed due to the diagnostic complexity, especially in patients who lack traditional stroke risk factors. This case study highlights a diagnostically challenging presentation of CVST in a middle-aged woman presenting with an atypical headache and no obvious vascular risk factors. Initial non-contrast computed tomography (CT) imaging revealed hemorrhagic infarction, but the diagnosis was only confirmed following CT venography. The case emphasizes the importance of early venous imaging in stroke presentations that exhibit atypical features. We discuss risk factors, the role of direct oral anticoagulants (DOACs), and evolving management strategies, including the screening for underlying prothrombotic conditions. Improved awareness, prompt imaging, and individualized anticoagulation strategies are key to optimizing outcomes in CVST. This case underscores the need for early recognition and multidisciplinary care.

## Introduction

Cerebral venous sinus thrombosis (CVST) is defined as a thrombosis situated within the dural venous sinuses or cerebral veins. It can cause impaired cerebral venous drainage, increased intracranial pressure, and potentially venous infarction or hemorrhage. CVST is a rare but potentially life-threatening cause of stroke. Although CVST accounts for only 0.5-1% of all strokes, its clinical significance is considerable due to the potential for full recovery with timely diagnosis and treatment [1,2].

Unlike arterial strokes, CVST predominantly affects younger individuals, with a noticeable female preponderance [3]. This is largely attributed to gender-specific risk factors such as pregnancy, the puerperium, and the use of hormonal contraceptives. However, numerous additional risk factors have been identified, such as inherited or acquired thrombophilias, malignancy, systemic infections, autoimmune conditions, and, more recently, COVID-19 infection and vaccine-induced immune thrombotic thrombocytopenia (VITT) [2,3]. While several risk factors are well-recognized, many patients may present without clear predisposing factors, which further adds to the diagnostic complexity of CVST [3].

The clinical presentation of CVST is highly variable and often non-specific [1]. Headache is the most frequent symptom, reported in up to 90% of patients, and may mimic migraine or tension-type headache. Other manifestations include focal neurological deficits (e.g., motor weakness, visual or speech or sensory disturbances), seizures, altered consciousness, and signs of raised intracranial pressure [2,4,5]. Because of this variability and lack of specificity, diagnosis can be challenging, particularly in emergency or resource-limited settings [1].

Imaging is essential for accurate diagnosis. A non-contrast computed tomography (CT) of the brain may show indirect signs such as cerebral edema, hemorrhagic infarction, or a hyperdense sinus, but it is often inconclusive [2,4]. Therefore, confirmatory imaging with CT venography (CTV) or magnetic resonance (MR) venography (MRV) is necessary to visualize the occluded sinuses directly [2,4]. According to the latest American Heart Association guidelines, venous imaging should be considered early in patients with unexplained neurological symptoms, especially in those without traditional vascular risk factors [2].

Below, we present a diagnostically challenging case of CVST in a middle-aged woman without typical vascular risk factors, emphasizing the need for early imaging and heightened clinical suspicion to identify CVST promptly.

## Case presentation

Presenting complaint

A 48-year-old woman presented to the emergency department (ED) of a district general hospital with a sudden-onset, severe bilateral frontal headache, rated 9/10 in intensity, associated with chills and three episodes of vomiting. She was witnessed becoming unresponsive briefly while walking on a golf course, during which she was noted to be rolling on the ground. She reported a similar episode of sudden-onset severe headache three days earlier, associated with photophobia and vomiting. She denied any fever, neck stiffness, rash, or head injury. She also denied any recent illnesses, unwell contacts, or similar episodes in the past.

Past medical history

Her past medical history included migraines, treated extrapulmonary tuberculosis, two miscarriages, and fertility issues. She had no history of hypertension, previous blood clots, weight changes, bleeding, or use of oral contraceptive pills and hormone replacement therapy. She did not smoke, drink, or use recreational drugs. She had no family history of thrombosis.

Examination and imaging

**Figure 1:**
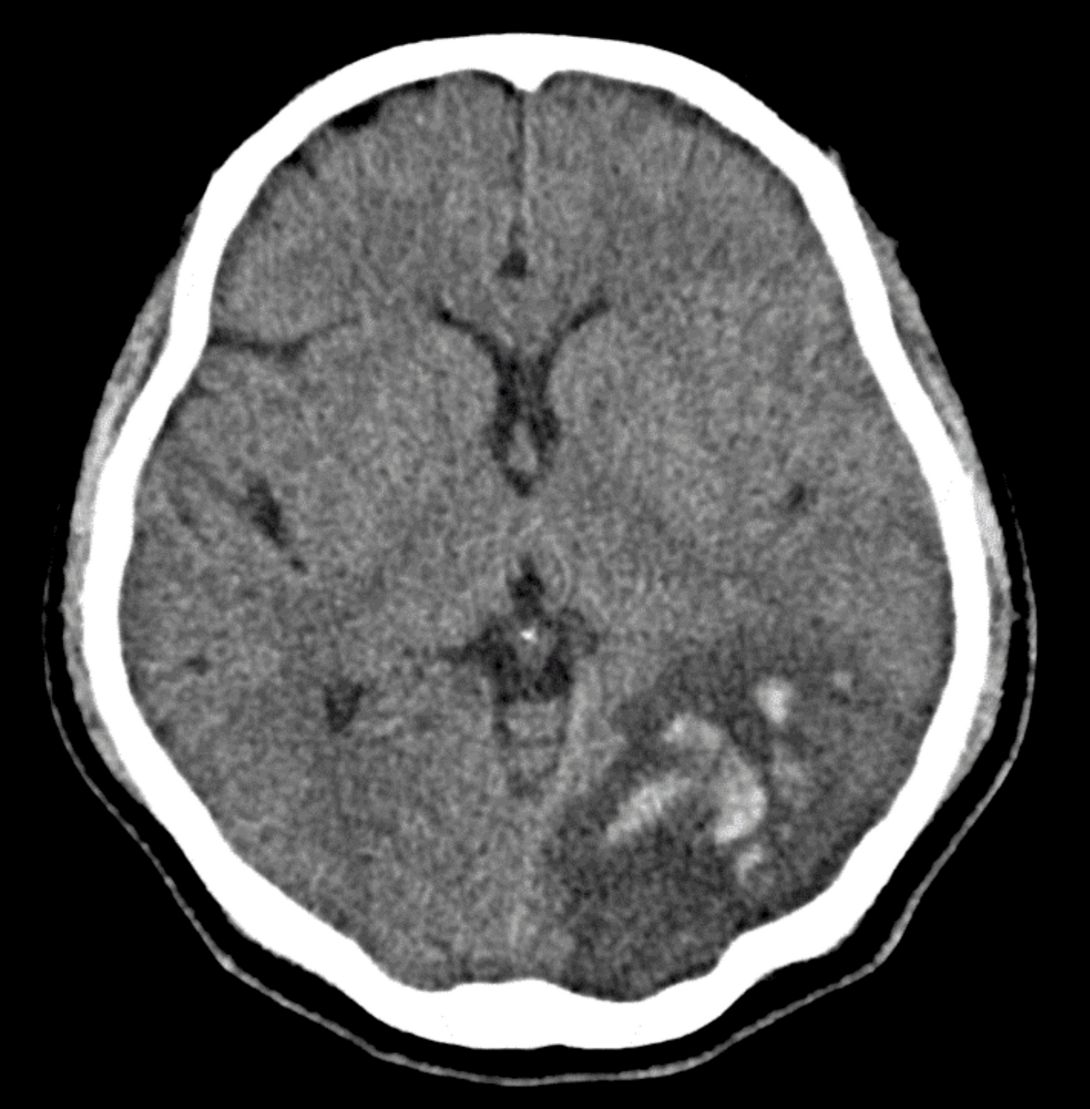
Axial non-contrast CT of the head demonstrating cerebral ischemia and foci of hemorrhage CT of the head showing a large area of parenchymal low density in the left parieto-occipital lobe. This is causing loss of grey-white matter differentiation, most likely due to subacute ischemia. There are also areas of associated hyperdensity which suggest acute parenchymal hemorrhage. CT: computed tomography

**Figure 2:**
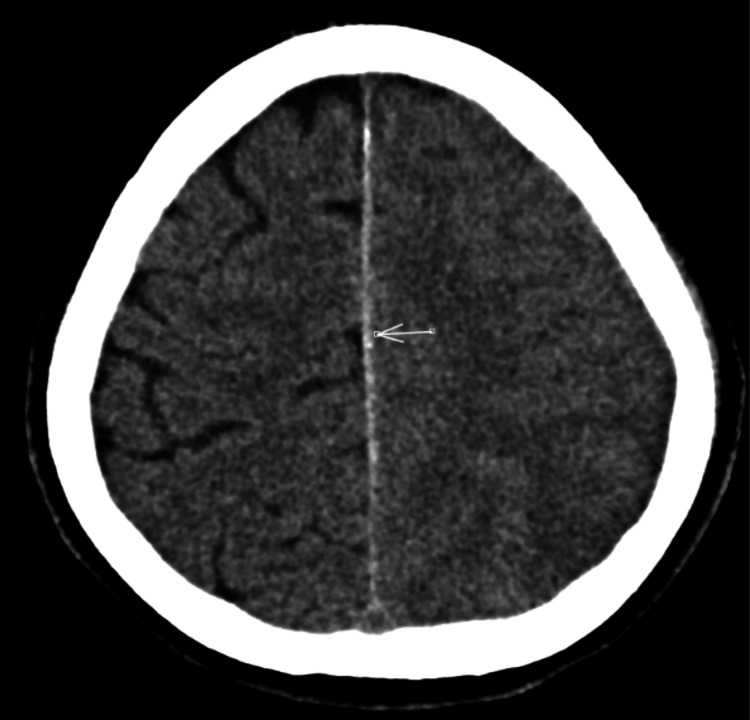
Axial non-contrast CT of the head demonstrating sulcal effacement CT of the head showing a mass effect within the left cerebral hemisphere causing sulcal effacement and a slight midline shift to the right. CT: computed tomography

Clinical examination revealed a fully conscious patient with normal power, tone, coordination, and sensation throughout the upper and lower limbs. There were no cranial nerve deficits, cerebellar signs, or gait ataxia. Examination of the cardiac, respiratory, and abdominal systems was unremarkable. The non-contrast CT of the brain showed a subacute left parieto-occipital infarct with hemorrhagic transformation (Figure 1), diffuse left cerebral edema, and acute left parafalcine and tentorial subdural hematomas (Figure 2).

**Figure 3:**
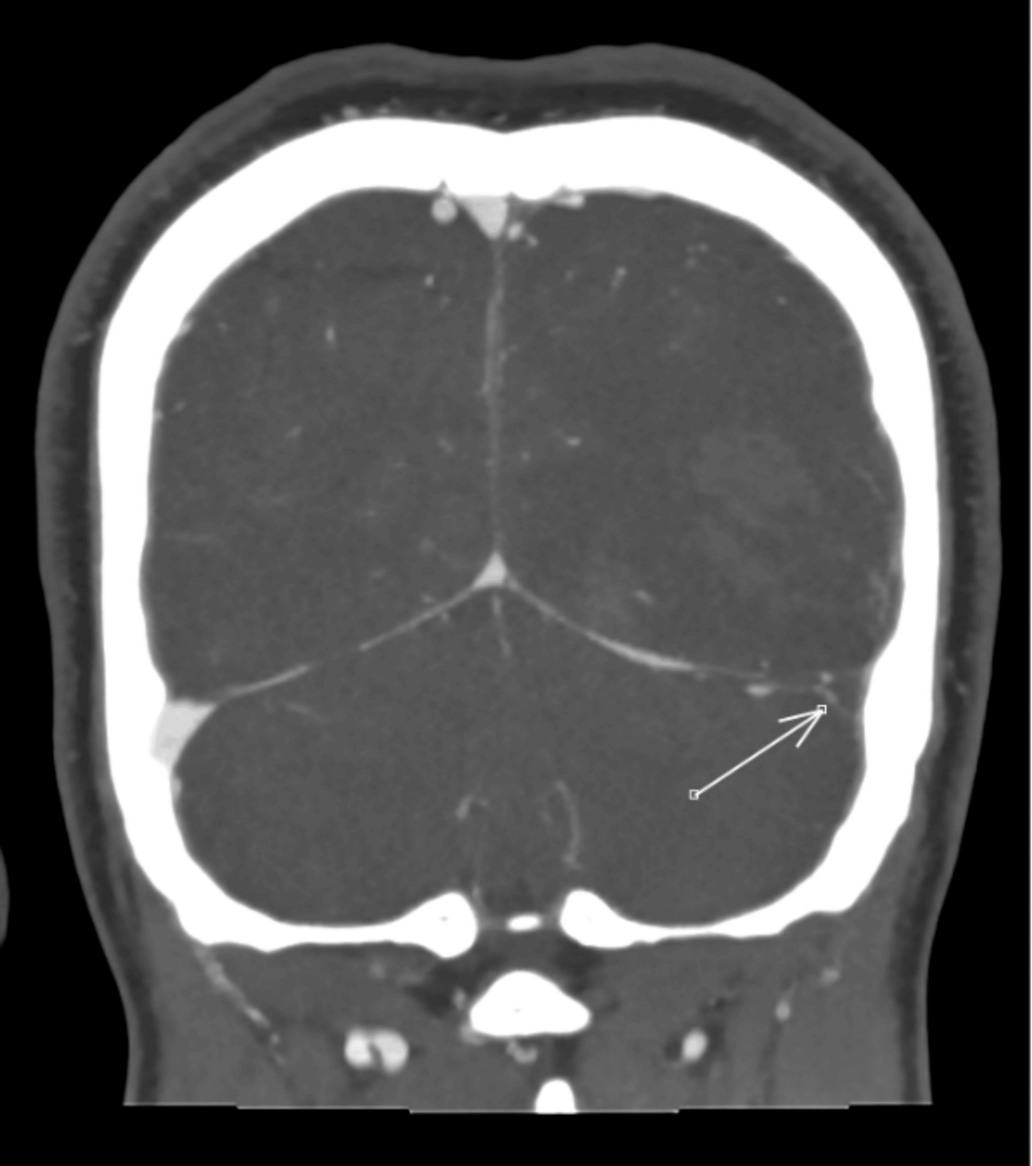
Coronal CT venogram demonstrating cerebral venous sinus thrombosis CT venogram demonstrating a filling defect within the left transverse sinus in keeping with cerebral venous sinus thrombosis. CT: computed tomography

**Figure 4:**
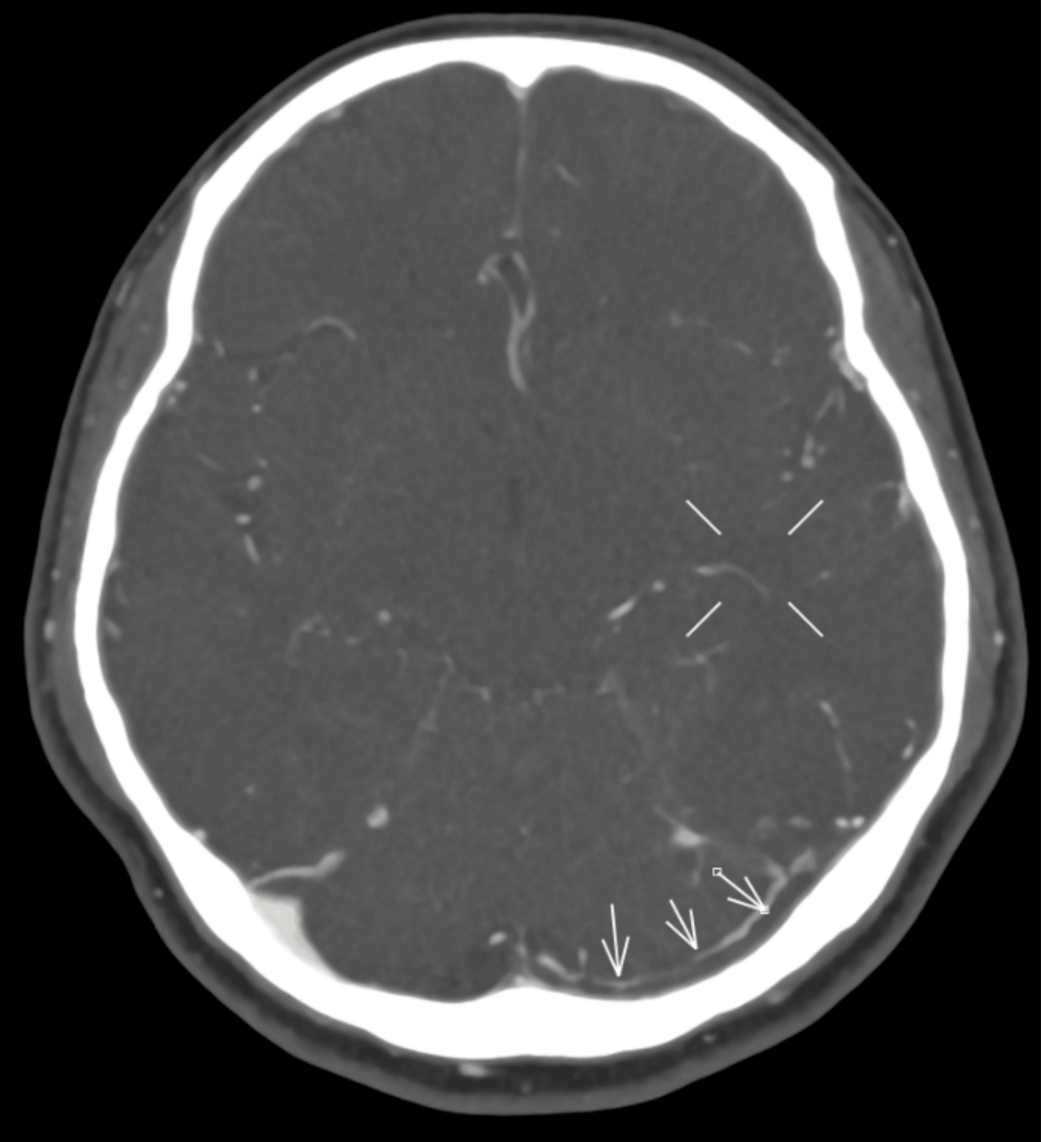
Axial CT venogram demonstrating cerebral venous sinus thrombosis CT venogram demonstrating a filling defect within the left transverse sinus in keeping with cerebral venous sinus thrombosis. CT: computed tomography

A discussion was made with the neurosurgeon, who advised a CT cerebral angiogram and venogram to investigate for vascular lesions or venous thrombosis, given the young age and no pre-existing hypertension. The cerebral angiogram revealed evidence of a hemorrhagic infarct and suggested a possible venous infarct. The CT venogram confirmed CVST involving the left transverse and sigmoid sinuses (Figures 3-4).

Blood tests showed iron deficiency anemia and slightly low vitamin B12, both of which were corrected. Her platelet count, clotting profile (prothrombin time, international normalized ratio, activated partial thromboplastin time, activated partial thromboplastin time ratio, and fibrinogen), thyroid function test, renal function, liver function, and celiac screen were normal. The CT of the chest, abdomen, and pelvis excluded malignancy.

Case management

The patient was commenced on low-molecular-weight heparin (enoxaparin 1 mg/kg subcutaneously every 12 hours) and later changed to dabigatran (150 mg orally twice daily), with follow-up arranged in the thrombosis clinic. A full thrombophilia screen, including antiphospholipid antibodies, paroxysmal nocturnal hemoglobinuria (PNH) screen, and myeloproliferative neoplasm (MPN) panel, was initiated. The patient was found to have raised anticardiolipin IgM, giving a high suspicion for antiphospholipid syndrome. She has been referred to the hematology clinic for further work-up. 

## Discussion

The main pillars of management for CVST are (1) anticoagulation, (2) management of underlying causes, and (3) consideration of invasive measures (e.g., endovascular thrombolysis or thrombectomy) [3]. 

Anticoagulation

Systemic anticoagulation, to arrest the thrombotic process (e.g., low-molecular-weight heparin or unfractionated heparin), is the first-line treatment for CVST [6]. This is endorsed by the European Federation of Neurological Societies (EFNS) guidelines as a safe treatment, even in the presence of hemorrhagic infarcts [7]. After a period of lead-in parenteral anticoagulation, it is then reasonable to transition onto oral anticoagulation (e.g., direct oral anticoagulant (DOAC) or vitamin K antagonist). There is no current evidence with a safe strategy for timings; however, in the majority of cases, patients are switched to DOACs within 5-15 days of low-molecular-weight heparin initiation [2]. There is no benefit of warfarin over DOACs, with similar rates of venous thromboembolism (VTE) recurrence and major hemorrhage [8-11]. 

The duration of this treatment is determined according to the underlying risk factors. The American Heart Association and American Stroke Association guidelines recommend 3-6 months in provoked CVST and 6-12 months in unprovoked CVST. Patients with recurrent CVST, severe thrombophilia, provoked CVST, or VTE following CVST may require lifelong anticoagulation [2]. 

In our case study, the patient was treated initially with low-molecular-weight heparin, and then, after clinical improvement, this was switched to dabigatran. This DOAC choice was in part due to the availability of a reversal agent, ease of administration, and lack of monitoring required. This was also deemed a practical choice given her stroke had been associated with foci of hemorrhage within the area of ischemia. 

Invasive management measures

For patients with CVST who deteriorate despite anticoagulant therapy, more invasive treatment measures may need to be considered. In some specialist tertiary centers, a thrombotic agent (e.g., urokinase) can be administered directly into the sinuses, alongside mechanical thrombo-aspiration, in an attempt at endovascular thrombolysis/thrombectomy. Another lifesaving therapeutic approach is decompressive craniectomy [12]. 

Risk factors for CVST

Extensive investigations are required for patients with CVST to rule out underlying risk factors. Risk factors can include rare hematological disorders (such as PNH and MPN), prothrombotic conditions (such as factor V Leiden and antithrombin III deficiency), and infections (e.g., human immunodeficiency virus (HIV) and tuberculosis) [6]. Screening blood tests required can include the following: PNH panel, factor V Leiden, protein C and S level, antithrombin levels and prothrombin gene mutation, antiphospholipid antibodies (anticardiolipin and beta-2 glycoprotein), and MPN screening. 

In our case, the patient was found to have raised anticardiolipin IgM. Alongside her history of recurrent miscarriages and a confirmed CVST, this made us highly suspicious of antiphospholipid syndrome. Therefore, she has been referred to the hematology clinic for further work-up, including repeating anticardiolipin IgM and IgG antibody levels.

## Conclusions

The diagnosis of CVST remains a challenge given the lack of specific clinical signs, vague diagnostic criteria, and difficulties in obtaining timely imaging. To improve diagnostic accuracy, clinicians should maintain a low threshold to obtain venous imaging for CVST in young patients presenting with headaches that are atypical, or progressively worsening, with a combined history of recent systemic illness or known prothrombotic conditions. Early venogram imaging is essential to allow for timely diagnosis and treatment. 
